# Beyond arbitrium: identification of a second communication system in Bacillus phage phi3T that may regulate host defense mechanisms

**DOI:** 10.1038/s41396-020-00795-9

**Published:** 2020-10-07

**Authors:** Charles Bernard, Yanyan Li, Philippe Lopez, Eric Bapteste

**Affiliations:** 1 Institut de Systématique, Evolution, Biodiversité (ISYEB), Sorbonne Université, CNRS, Muséum National d’Histoire Naturelle, Campus Jussieu, Bâtiment A, 4eme et. Pièce 429, 75005 Paris, France; 2 Unité Molécules de Communication et Adaptation des Micro-organismes (MCAM), CNRS, Muséum National d’Histoire Naturelle, CP 54, 57 rue Cuvier, 75005 Paris, France

**Keywords:** Bacteriophages, Viral genetics

## Abstract

The evolutionary stability of temperate bacteriophages at low abundance of susceptible bacterial hosts lies in the trade-off between the maximization of phage replication, performed by the host-destructive lytic cycle, and the protection of the phage-host collective, enacted by lysogeny. Upon *Bacillus* infection, Bacillus phages phi3T rely on the “arbitrium” quorum sensing (QS) system to communicate on their population density in order to orchestrate the lysis-to-lysogeny transition. At high phage densities, where there may be limited host cells to infect, lysogeny is induced to preserve chances of phage survival. Here, we report the presence of an additional, host-derived QS system in the phi3T genome, making it the first known virus with two communication systems. Specifically, this additional system, coined “Rapφ-Phrφ”, is predicted to downregulate host defense mechanisms during the viral infection, but only upon stress or high abundance of *Bacillus* cells and at low density of population of the phi3T phages. Post-lysogenization, Rapφ-Phrφ is also predicted to provide the lysogenized bacteria with an immediate fitness advantage: delaying the costly production of public goods while nonetheless benefiting from the public goods produced by other non-lysogenized *Bacillus* bacteria. The discovered “Rapφ-Phrφ” QS system hence provides novel mechanistic insights into how phage communication systems could contribute to the phage-host evolutionary stability.

## Introduction

Temperate bacteriophages are viruses that can infect their bacterial hosts either through the host-destructive lytic cycle or the non-destructive lysogenic cycle. During the latter stage, the phage replicates as part of the bacterial genome as a “prophage” and confers immunity to a second infection upon the lysogenized bacterium [1, 2]. Lysogeny, therefore, is a strategy that protects the phage-host collective [3].

Remarkably, Erez et al. observed that upon *Bacillus subtilis* infection, Bacillus phage phi3T (phi3T) relies on an endogenous quorum sensing (QS) system called “arbitrium” to orchestrate the lysis-to-lysogeny transition, when phages become abundant and the susceptible hosts left to infect are likely to be few [4].

Here, we report the discovery of a second communication system, termed “Rapφ-Phrφ”, in the genome of phi3T, now the first known virus with two communication systems. Rapφ-Phrφ is the first known phage-encoded QS system predicted to regulate bacterial mechanisms, and in both a phage- and a host-density dependent manner.

## Results and discussion

**Fig. 1:**
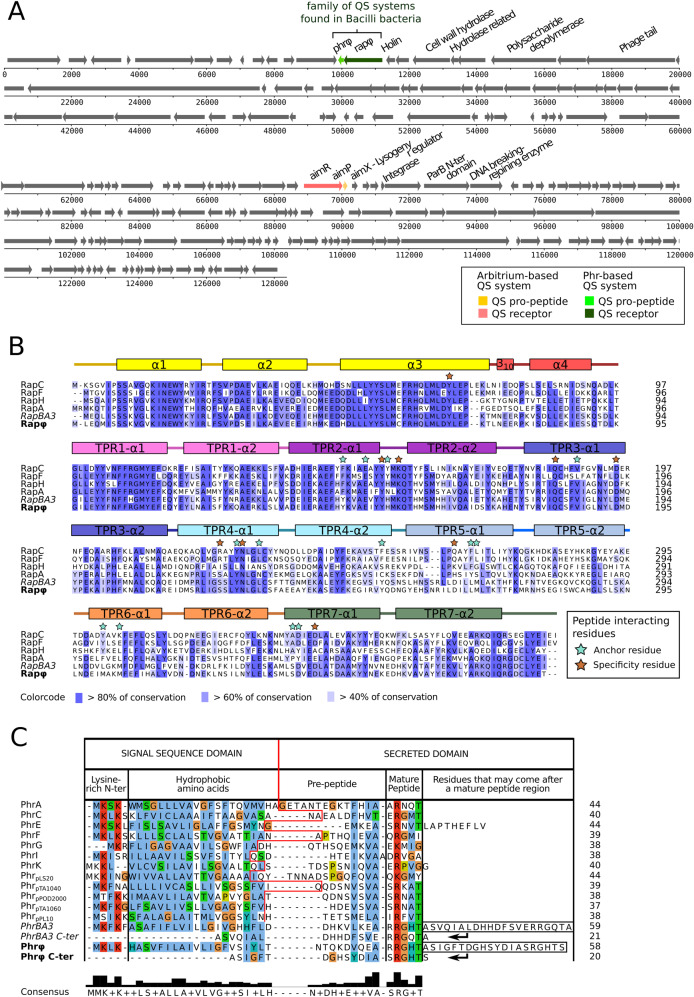
Characterization of the Rapφ-Phrφ QS system in Bacillus phage phi3T. **a** Position of the *rapφ-phrφ* and of the arbitrium (*aimR-aimP*) QS systems in the genome of phi3T. On this genomic map, arrows pointing towards the right correspond to genes on the “+” DNA strand whereas arrows pointing towards the left correspond to genes on the “−“ strand. The position of each gene in the genome of phi3T is indicated by the horizontal axis (number of base pairs). The genes belonging to a QS system are in color. The *aimX* regulator of lysogeny is located downstream from *aimP*. The genes in the vicinity of each QS system are functionally annotated, whenever it was possible. **b** Multiple sequences alignment of the RapA, C, F and H proteins (*B. subtilis* str. 168) for which the structure has been resolved in Gallego del Sol and Marina [7] alongside RapBA3 (prophage of *B. velezensis* FZB42) and Rapφ (Bacillus phage phi3T). The N-terminal region of the Rap proteins is involved in the interaction with the ComA-P and/or Spo0F-P response regulators whereas the C-terminal region (TPR repeats) is involved in the interaction with the Phr_mature_ QS peptide. Residues are colored according to the “% identity” colorcode. The residues that have been identified by Gallego del Sol and Marina as involved in the interaction with the QS peptide are annotated by stars, and brown stars specifically highlight the residues responsible for the specificity of Rap towards its cognate Phr_mature_ peptide. **c**. Multiple sequences alignment of the canonical Phr proteins considered in Pottathil et al [10] alongside the full PhrBA3 protein, the C-terminus of PhrBA3 that follows the first PhrBA3_mature_ region (RRGHT), the full Phrφ protein, and the C-terminus of Phrφ that follows the first Phrφ_mature_ region (RRGHT). Residues are colored according to the “Clustal” colorcode (see: http://www.jalview.org/help/html/colourSchemes/clustal.html). The red line in the middle of the alignment separates the N-terminal signal sequence domain from the C-terminal secreted domain of Phr proteins, as annotated by Pottathil et al. The sequences of the different QS pentapeptides are indicated by the “Mature Peptide” column.

In addition to arbitrium, we report that Bacillus phage phi3T encodes a second QS system, coined “Rapφ–Phrφ”, from the Rap-Phr family of *Bacillus* bacteria [5, 6] (Fig. 1a). The Rapφ intracellular QS receptor (NCBI accession APD21157.1) is a 379aa long protein that shows 48% sequence identity over its entire length with RapC, a well-characterized member of the Rap protein family [7, 8] (Fig. 1b). Rapφ harbors tetratricopeptide repeats (TPR) (Pfam PF13181.6 and PF13424.6 HMM profiles), typically involved in the binding of QS peptides [9] (Fig. 1b). Phrφ (NCBI accession APD21156.1) is a 58aa long protein that exhibits all the characteristics of Rap cognate Phr pre-pro-peptides [10], both in terms of sequence composition (Fig. 1c) and genetic organization, with the *phr* gene directly downstream of *rap*, on the same DNA strand (Fig. 1a). Phrφ is predicted to be secreted via the SEC-translocon by SignalP-5.0 [11] (likelihood of 0.76) and harbors two characteristic regions that might be processed by extracellular proteases into the mature R-R-G-H-T and/or S-R-G-H-T QS signal pentapeptide(s) (Fig. 1c). Although non-canonical, the presence of two regions that are eventually matured into QS peptides was reported in many other functional Phr proteins [5]. The *rapφ-phrφ* locus forms an operon, directly flanked downstream and upstream by intrinsic terminators (Table S1). The mapping of RNA-seq reads sequenced during the infection of *Bacillus* by phi3T [4] to the phi3T genome indicated that *rapφ* and *phrφ* are expressed upon *Bacillus* infection (Fig. S1).

In the *Bacillus* genus, Rap-Phr systems are diverse, often strain specific and subject to horizontal transfers [5, 6]. We identified 2360 homologs of the Rapφ-Phrφ QS systems in the complete genomes of Firmicutes available on the NCBI, including 333 predicted by Phaster [12] to belong to prophages, suggesting that *rap-phr* QS systems are widely used and horizontally transferred by phages (Table S2 and Fig. S2). Two of the QS systems carried by prophages (RapBA3-PhrBA3 in *B. velezensis* FZB42 and RapBL5-PhrBL5 in *B. licheniformis* ATCC14580) have previously been functionally tested via a dedicated synthetic construct in *B. subtilis* strain PY79 (although it was then unknown that these systems belong to prophages) [5].

**Fig. 2:**
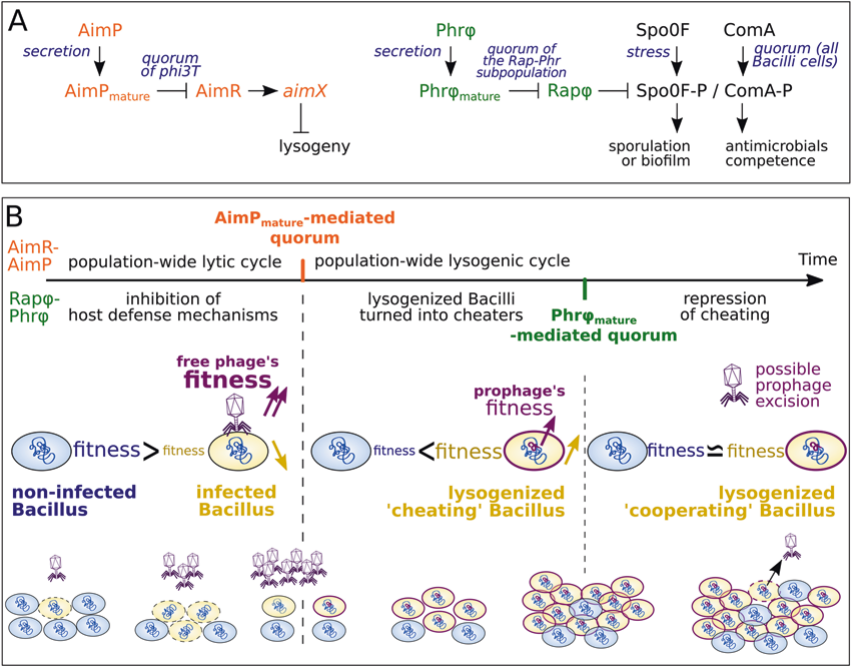
Hypotheses on the mechanism of Rapφ-Phrφ, on its synergy with the arbitrium system, and on the evolutionary advantages for phi3T to own both systems. **a** Wiring diagrams of the characterized mechanism of the arbitrium system (left) and of the predicted mechanism of Rapφ-Phrφ (right). Upon *Bacillus* infection, AimP and Phrφ are predicted to be exported in the medium by the SEC-translocon and cleaved by extracellular peptidases into mature QS peptides that accumulate in the medium as phi3T replicates. At low densities of phi3T, the unbound AimR QS receptor activates the expression of *aimX* encoding the negative regulator of lysogeny, which ensures a population-wide lytic cycle. At high densities of phi3T, AimP_mature_ reaches a threshold extracellular concentration and is consistently imported by neighboring cells via the Opp permease. Within infected  *Bacillus* cells, AimP_mature_ inhibits AimR and alleviates lysogeny inhibition. A population-wide lysogeny is then triggered. At low densities of phi3T, the unbound Rapφ QS receptor is predicted to inhibit the ComA-P and Spo0F-P response regulators of *Bacillus* (if the host is stressed or if the entire *Bacillus* population is at high density), which might downregulate host defense mechanisms. Either at high densities of phi3T or at high densities of the lysogenized subpopulation, Phr_mature_ is predicted to inhibit Rapφ, which might reactivate the ComA-P and Spo0F-P pathways of the host. **b** A timeline is drawn on the top of the panel; the orange and the green vertical marks indicate when the quorums mediated by AimP_mature_ and by Phr_mature_ are proposed to intervene. The different mechanisms regulated by the arbitrium QS system over time are indicated on the topside of the timeline whereas the ones predicted to be regulated by Rapφ-Phrφ are indicated on the bottom side. The predicted variations of density of the different subpopulations (phages phi3T, non-infected, infected and lysogenized *Bacillus* bacteria) over time are shown in the bottom of the panel. Their relative predicted fitness over time is shown in the middle of the panel. Before the AimP_mature_-mediated quorum, phi3T quickly replicates at the expense of *Bacillus* during the lytic cycle. Meanwhile, Rapφ might enhance the efficiency of the lytic cycle by downregulating host defense mechanism when *Bacillus* cells are abundant. When phages become abundant and when there may be limited host cells to infect, the AimP_mature_-mediated quorum triggers the host-protective lysogenic cycle and phi3T phages are internalized as prophages. The arbitrium QS system thus maximizes the time spent in the lytic cycle before it becomes a threat for the phage-host collective. After lysogeny, the Rapφ protein of phi3T prophages might enhance the fitness of the prophage-host collective, by conferring an immediate fitness advantage on the freshly lysogenized subpopulation, likely afflicted by the previous lytic cycle. Indeed, whenever the entire *Bacillus* population would be at high densities and would transduce the signal for the production of public goods, the ComA-P and Spo0F-P inhibitions via Rapφ might provide the lysogenized subpopulation with the means to cheat (delay the costly production of biofilm molecules and antimicrobials) until this lysogenized subpopulation reaches again a substantial cellular density, as reflected by a threshold concentration of Phrφ_mature_.

At low densities of the *Bacillus* subpopulation that either expresses the cloned *rapBA3* or *rapBA5* receptors, the Rap protein was experimentally demonstrated to strongly inhibit the Spo0F-P and ComA-P response regulators [5]. The Spo0F-P pathway leads to biofilm formation or sporulation [13, 14], shown to elicit the lytic cycle of certain bacteriophages [15, 16]. The ComA-P pathway triggers competence and the production of antimicrobials [17] that might contribute to the defense against phages. As a Rap-Phr expressing *Bacillus* subpopulation grows, the secreted Phr_mature_ peptide accumulates in the medium. At high concentrations, PhrBA3_mature_ and PhrBL5_mature_ are internalized by the Opp permease and demonstrably sequester their cognate Rap receptor, which, in turn, alleviates the inhibition of ComA-P, Spo0F-P and their target pathways [5]. Therefore, there is evidence that Rap-Phr systems of prophages can delay, in *Bacillus*, defense mechanisms (antimicrobials, sporulation) and the production of public goods (biofilm molecules, antimicrobials) until the *Bacillus* subpopulation expressing the prophage QS system reaches a substantial cellular density (Figs. 2a and S3). Importantly, Rapφ-Phrφ is highly similar to RapBA3-PhrBA3. Rapφ and RapBA3 yield 77% sequence identity at full mutual coverage and harbor the exact same residues that account for the QS peptide specificity (Fig. 1b). Consistently, Phrφ presents a mature peptide region (R-R-G-H-T), identical to the PhrBA3_mature_ QS peptide shown to inhibit RapBA3 [5] (Fig. 1c).

On these bases, we predict that Rapφ-Phrφ is likely to be functional and that Rapφ can thus downregulate host defense mechanisms via Spo0F-P and ComA-P inhibition, enhancing the efficiency of the phi3T lytic cycle. Importantly, Rapφ regulation of host processes is predicted to occur only at low concentrations of Phrφ_mature_ and when Spo0F is phosphorylated upon stress or ComA during the QS response of the entire, mixed population of *Bacillus* cells [13, 18, 19] (see refs. [20–23] for the potential advantages for a phage to inhibit the host’s QS response) (Figs. 2 and S4). Hence, when defense pathways are triggered at high densities of *Bacillus* bacteria, phi3T phages might “disarm” their host only at low densities of phages, when the phage-host collective is not yet endangered. Meanwhile, the arbitrium system of phi3T ensures a fast-replication through the lytic cycle when phages are rare. When phages become abundant, it triggers a population-wide, host-protective lysogeny [4]. Particularly, if the quorum mediated by arbitrium were reached before that of Phrφ_mature_, the Rapφ protein of phi3T prophages could delay the production of public goods in their freshly lysogenized hosts until the lysogenized subpopulation, likely afflicted by the previous lytic cycle, reaches a substantial density [19]. Rapφ-Phrφ could hence momentarily turn lysogenized *Bacillus* bacteria into cheaters, enhancing both the fitness of these cells and of the phi3T prophages. Interestingly, one prophage of *B. velezensis* SQR9 carried both the arbitrium and the Rap-Phr QS systems, as in phi3T (Fig. S2B), further supporting that owning both communication systems might be advantageous for a temperate bacteriophage.

## Supplementary information

Supplementary Information

Table S1

Table S2

## Data Availability

All data generated or analyzed during this study are included in this published article and its Supplementary information files. The genome of Bacillus phage phi3T can be accessed from the NCBI “Assembly” database with the “ASM260144v1” accession ID. The accession IDs for the protein sequences of Rapφ and Phrφ are “APD21157.1” and “APD21156.1”, respectively.
