# Supplementary material for: Beyond arbitrium: identification of a second communication system in Bacillus phage phi3T that may regulate host defense mechanisms
Source: ISME J. 2020 Oct 7;15(2):545–9. doi: 10.1038/s41396-020-00795-9 (PMC8027211; doi:10.1038/s41396-020-00795-9)
Supplement: Supplementary file 1 — Supplementary Information [file 41396_2020_795_MOESM1_ESM.pdf]

Figure S1

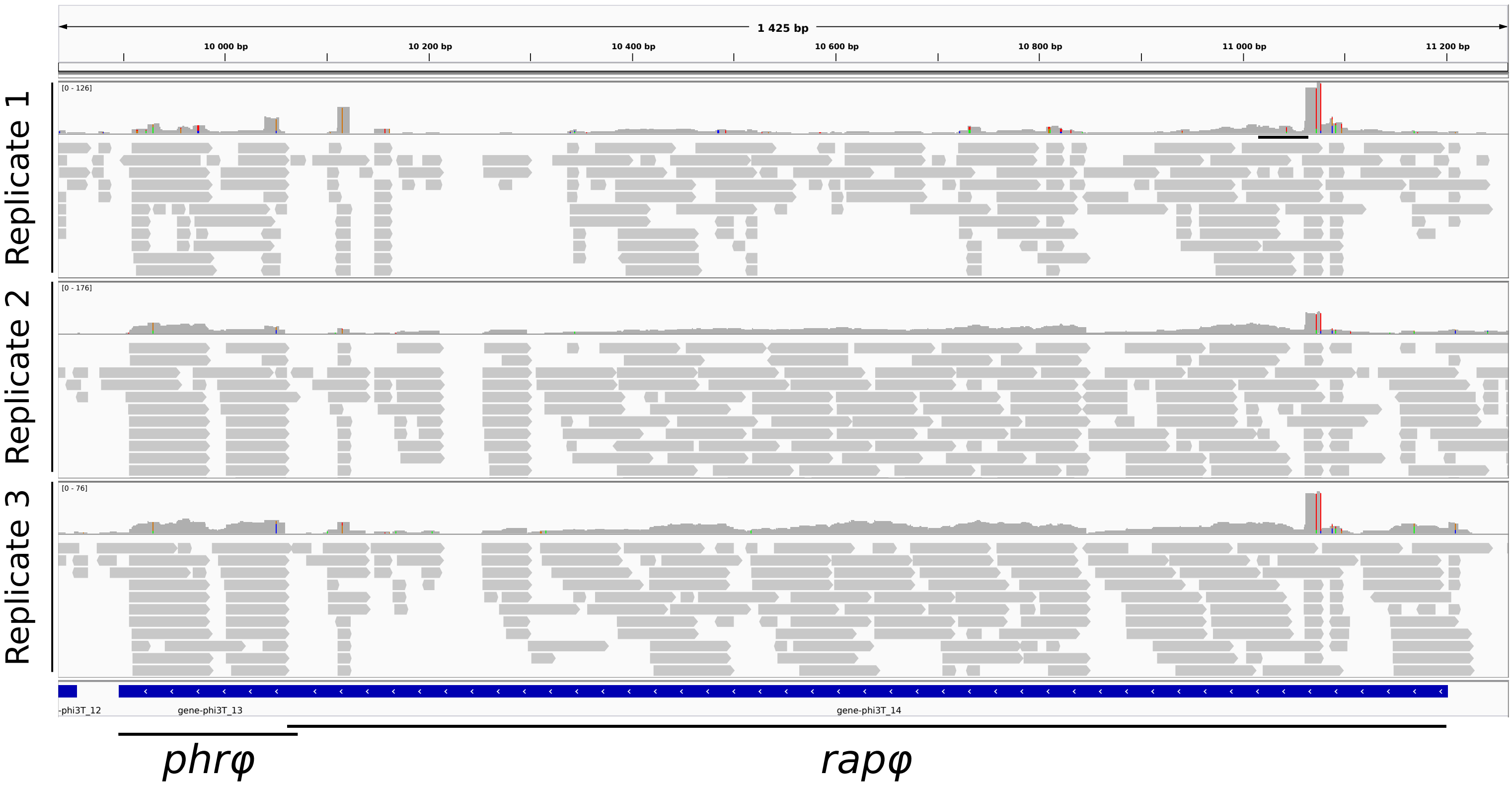
1 425 bp
10 000 bp
10 200 bp
10 400 bp
10 600 bp
10 800 bp
11 000 bp
11 200 bp
Replicate 1
[0 - 126]
Replicate 2
[0 - 176]
Replicate 3
[0 - 76]
-phi3T_12
gene-phi3T_13
gene-phi3T_14
phrφ
rapφ

Figure S1: Coverage profile and alignments of RNA-Seq reads to the *rapφ-phrφ* locus.

Each horizontal box corresponds to a replicate of RNA-seq reads, sequenced by Erez et al. (1) after 20min of infection of *Bacillus* str. BEST7007 by phage phi3T. For each replicate, the coverage profile is displayed at the top of the box, and the first 11 lines of the alignment at the bottom. The two segments at the bottom of the figure delineate the overlapping *phrφ* and *rapφ* genes.

Figure S2

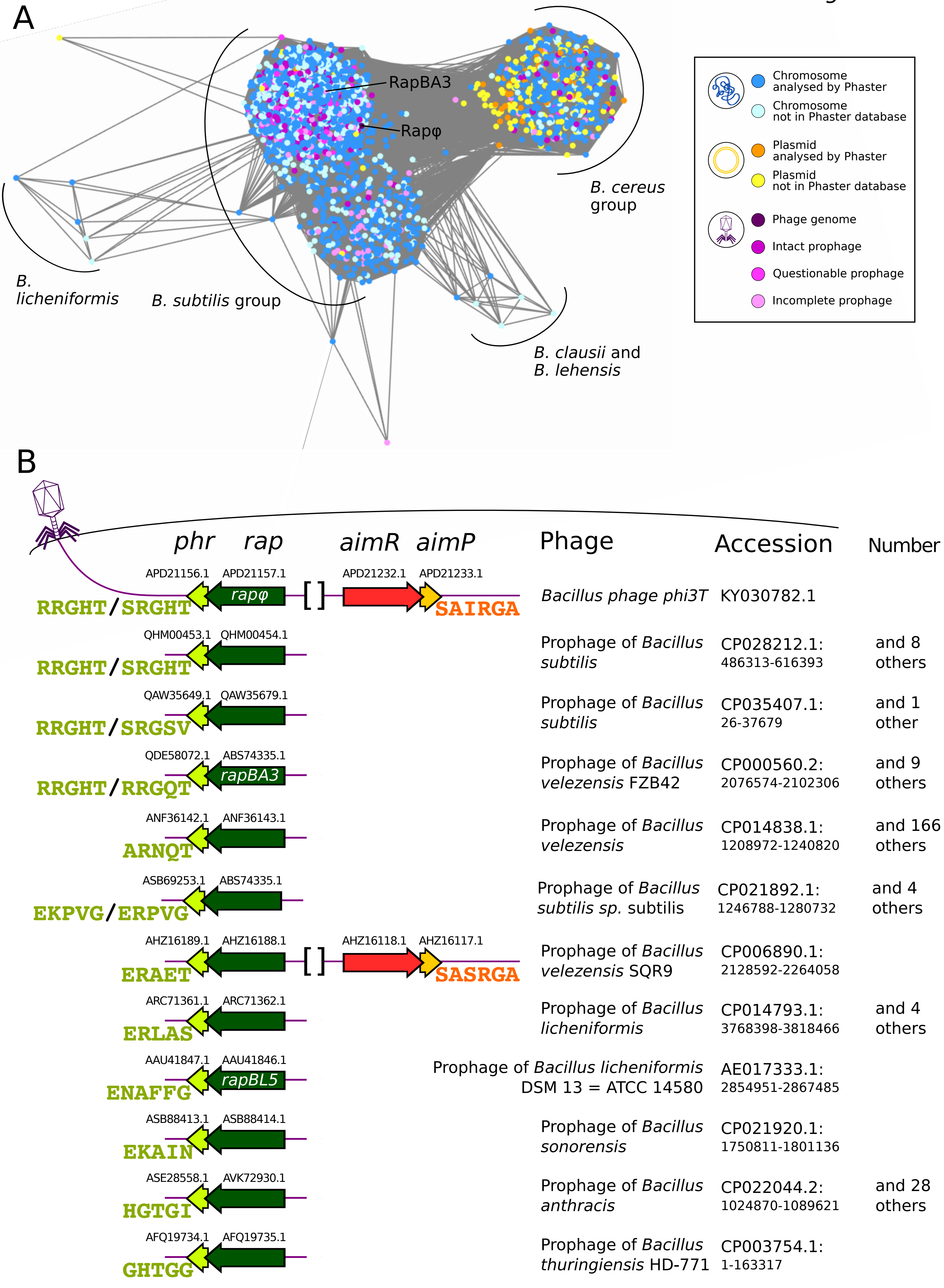

A
RapBA3
Rapφ
B. licheniformis
B. subtilis group
B. cereus group
B. clausii and B. lehensis
Chromosome analysed by Phaster
Chromosome not in Phaster database
Plasmid analysed by Phaster
Plasmid not in Phaster database
Phage genome
Intact prophage
Questionable prophage
Incomplete prophage
B
phr
rap
aimR
aimP
Phage
Accession
Number
APD21156.1
APD21157.1
rapφ
APD21232.1
APD21233.1
RRGHT/SRGHT
SAIRGA
Bacillus phage phi3T
KY030782.1
QHM00453.1
QHM00454.1
RRGHT/SRGHT
Prophage of Bacillus subtilis
CP028212.1: 486313-616393
and 8 others
QAW35649.1
QAW35679.1
RRGHT/SRGSV
Prophage of Bacillus subtilis
CP035407.1: 26-37679
and 1 other
QDE58072.1
ABS74335.1
rapBA3
RRGHT/RRGQT
Prophage of Bacillus velezensis FZB42
CP000560.2: 2076574-2102306
and 9 others
ANF36142.1
ANF36143.1
ARNQT
Prophage of Bacillus velezensis
CP014838.1: 1208972-1240820
and 166 others
ASB69253.1
ABS74335.1
EKPVG/ERPVG
Prophage of Bacillus subtilis sp. subtilis
CP021892.1: 1246788-1280732
and 4 others
AHZ16189.1
AHZ16188.1
AHZ16118.1
AHZ16117.1
ERAET
SASRGA
Prophage of Bacillus velezensis SQR9
CP006890.1: 2128592-2264058
ARC71361.1
ARC71362.1
ERLAS
Prophage of Bacillus licheniformis
CP014793.1: 3768398-3818466
and 4 others
AAU41847.1
AAU41846.1
rapBL5
ENAFFG
Prophage of Bacillus licheniformis DSM 13 = ATCC 14580
AE017333.1: 2854951-2867485
ASB88413.1
ASB88414.1
EKAIN
Prophage of Bacillus sonorensis
CP021920.1: 1750811-1801136
ASE28558.1
AVK72930.1
HGTGI
Prophage of Bacillus anthracis
CP022044.2: 1024870-1089621
and 28 others
AFQ19734.1
AFQ19735.1
GHTGG
Prophage of Bacillus thuringiensis HD-771
CP003754.1: 1-163317

Figure S2: Abundance and diversity of Rap-Phr systems in Bacillus' prophages.

A. Sequence similarity network of the Rap proteins that form a Rap-Phr system in the complete genomes of Firmicutes. Each node corresponds to a Rap protein and each edge between two nodes (two Raps) is supported by a sequence identity > 30% and a mutual coverage > 80%. The nodes are colored according to the type of DNA that encodes the Rap-Phr system, as displayed by the legend. Specifically, there are 1849 chromosomal Rap-Phr systems (including 1330 asserted by Phaster as not belonging to a prophage), 178 plasmidic Rap-Phr systems (including 43 asserted by Phaster as not belonging to a prophage), 1 Rap-Phr system in a phage genome (Rapφ-Phrφ) and 333 Rap-Phr systems predicted by Phaster as belonging to prophages (160 to intact prophages, 59 to questionable prophages and 114 to incomplete prophages).

B. Overview of the diversity of the Rap-Phr systems carried by prophages. Each line corresponds to the DNA of a phage or a prophage that encodes a Rap-Phr QS system and that may as well encode an arbitrium QS system. Each shown prophage is representative of a given type of Rap-Phr system, as determined by the sequence(s) of the predicted Phr_{mature} QS peptide(s). The number on the right side of each line gives the number of prophages that a displayed prophage is representative of. For each prophage, the figure shows the predicted sequence(s) of the Phr_{mature} and $AimP_{mature}$ QS peptide(s), the NCBI ID of each QS protein, the host bacterial genome and the coordinates of the prophage in this bacterial genome (predicted by Phaster). The RapBA3-PhrBA3 and the RapBL5-PhrBL5 QS systems that have previously been functionally validated by Even-Tov et al (2) are also indicated.

Figure S3

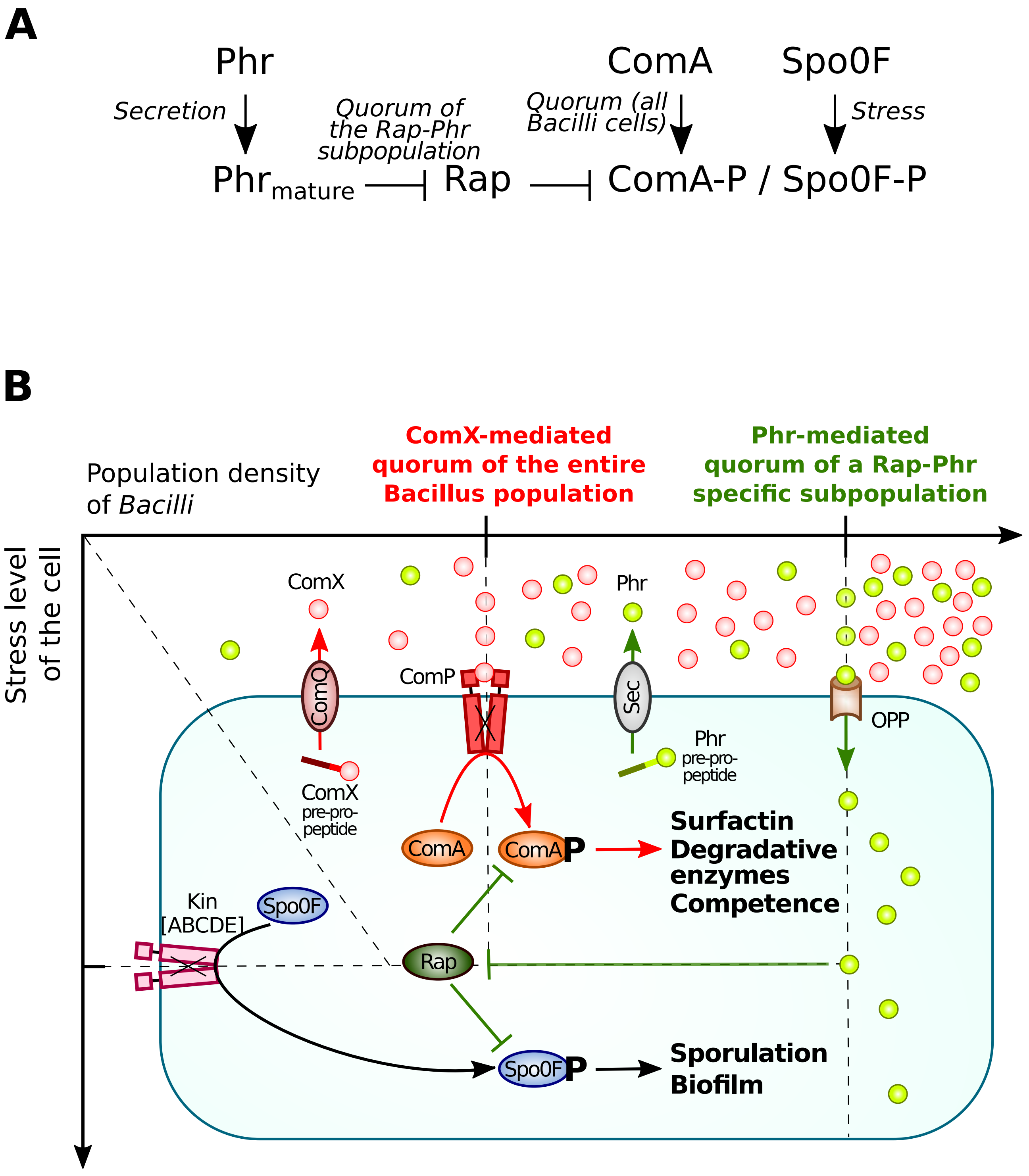
A
Phr
Secretion
Phr mature
Quorum of the Rap-Phr subpopulation
Rap
ComA
Quorum (all Bacilli cells)
Spo0F
Stress
ComA-P / Spo0F-P
B
Population density of *Bacilli*
ComX-mediated quorum of the entire Bacillus population
Phr-mediated quorum of a Rap-Phr specific subpopulation
Stress level of the cell
ComX
ComQ
ComX pre-pro-peptide
ComP
ComA
ComAP
Phr
Sec
Phr pre-pro-peptide
OPP
Surfactin
Degradative enzymes
Competence
Kin [ABCDE]
Spo0F
Rap
Spo0FP
Sporulation
Biofilm

Figure S3: Typical mechanism of a Rap-Phr QS system in *Bacillus* bacteria

A. Wiring diagram of the molecular mechanism of a Rap-Phr system.

B. Illustration of the molecular mechanism of a Rap-Phr system within a Bacillus cell. On the top hemisphere of the cell and from the left to the center, is schematized the ComX-based Quorum Sensing mechanism in *Bacillus* bacteria. The different steps of this mechanism are depicted by red arrows. The ComX pre-pro-peptide is exported in the medium and processed by ComQ to mature to $ComX_{mature}$ QS peptide. When the concentration of $ComX_{mature}$ reaches a threshold and reflects the quorum of the mixed population of Bacilli, it robustly binds to the ComP histidine kinase, which then transfers a phosphate to ComA, the response regulator of the ComX-based QS response. Once phosphorylated, ComA-P triggers the expression of genes related to competence and to the production of antimicrobials and degradative enzymes. From the topleft to the bottom of the cell is schematized the typical mechanism of the stress response. Upon receiving a stress stimulus, stress kinases phosphorylate the Spo0F protein, which thereupon initiates a phosphorelay chain through several intermediate proteins that eventually triggers sporulation. From the center to the right of the cell is schematized the typical mechanism of a Rap-Phr QS system. Green arrows highlight the different steps of this mechanism. Below the Phr-quorum, the intracellular Rap receptor antagonizes either or both of the ComA-P and Spo0F-P response regulators, either through dephosphorylation or physical sequestration. Meanwhile, the Phr protein is exported by the Sec secretion system and is cleaved by exopeptidases to produce a mature QS peptide. When the Phr_{mature} QS peptide reaches a threshold concentration, it is imported by the OPP permease, and further sequesters the Rap intracellular receptors, thereby releasing the inhibition of the ComA-P and Spo0F-P regulators. The quorum mediated by Phr_{mature} often intervenes after the quorum mediated by $ComX_{mature}$ because a Rap-Phr system is often expressed by a subpopulation of cells, unlike the ComX-ComP system which is expressed by all Bacilli.

Figure S4

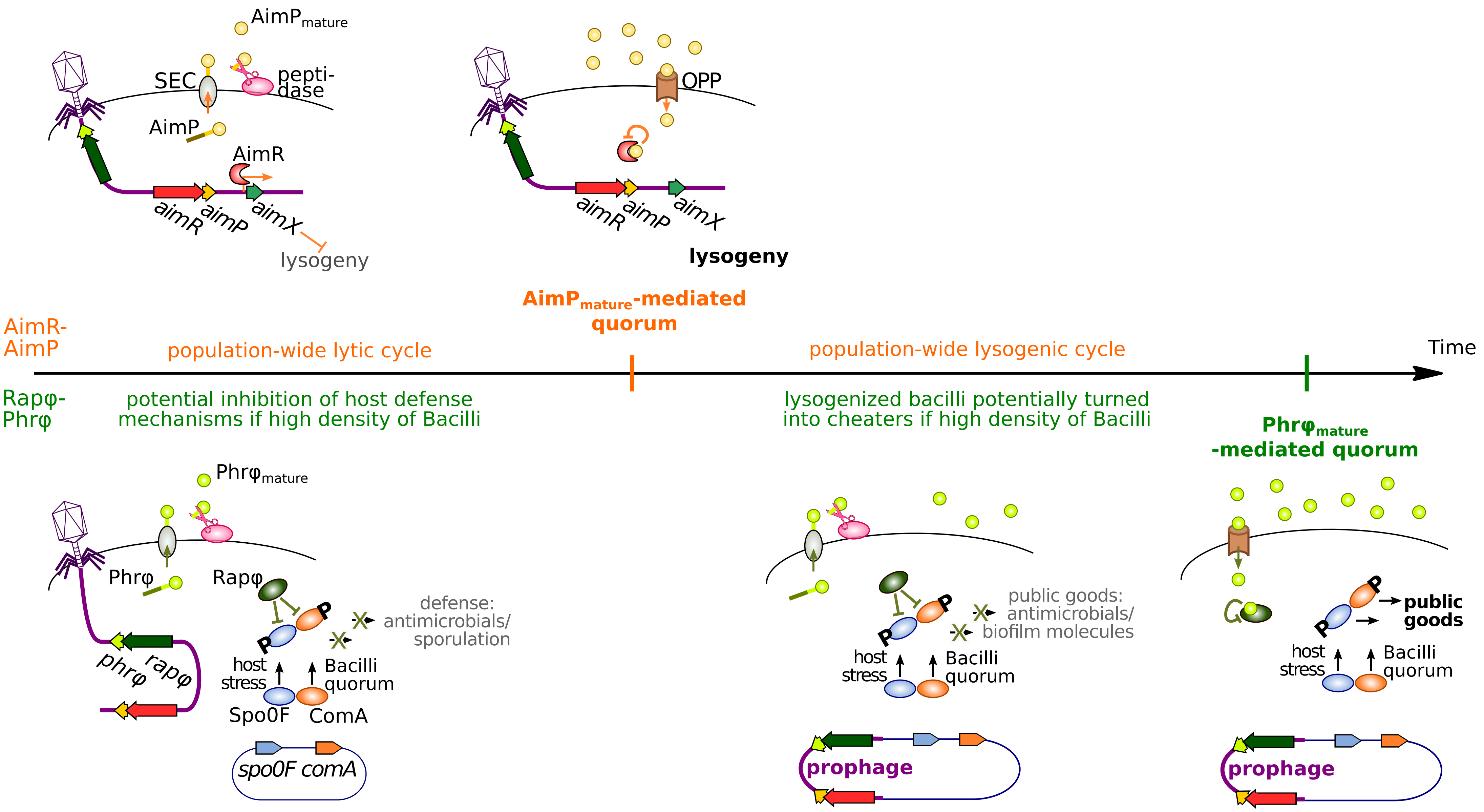

AimPmature
SEC
pepti-dase
AimP
AimR
aimR
aimP
aimX
lysogeny
OPP
aimR
aimP
aimX
lysogeny
AimPmature-mediated quorum
AimR-AimP
population-wide lytic cycle
population-wide lysogenic cycle
Time
Rapφ-Phrφ
potential inhibition of host defense mechanisms if high density of Bacilli
lysogenized bacilli potentially turned into cheaters if high density of Bacilli
Phrφmature -mediated quorum
Phrφmature
Phrφ
Rapφ
defense: antimicrobials/ sporulation
phrφ
rapφ
host stress
Bacilli quorum
Spo0F
ComA
spo0F comA
public goods: antimicrobials/ biofilm molecules
host stress
Bacilli quorum
prophage
public goods
host stress
Bacilli quorum
prophage

Figure S4: Mechanism of the arbitrium QS system and proposed mechanism of the Rapφ-Phrφ QS system in Bacillus phage phi3T.

On the top is displayed the characterized mechanism of the arbitrium QS system of phi3T (with the different steps controlled by the system colored in orange) (1). At low density of phages, unbound AimR indirectly inhibits lysogeny while the $AimP_{mature}$ peptide is secreted and accumulates in the medium. At high density of phages, $AimP_{mature}$ is imported by the Opp permease, binds to AimR, and alleviates lysogeny inhibition. Thereby, the arbitrium QS system ensures that the lytic cycle continues as long as the phages are few and the phage-host collective is not critically threatened by the phage infection. On the bottom is displayed the predicted mechanism of the Rapφ-Phrφ QS system of phi3T (with the different steps controlled by the system colored in green), in the proposed scenario where the quorum mediated by $AimP_{mature}$ is reached before the quorum mediated by $Phr\varphi_{mature}$. At low density of phages, Rapφ is predicted to enhance the efficiency of the phi3T lytic cycle. Indeed, Rapφ is predicted to inhibit ComA and Spo0F and their target pathways linked to defense mechanisms in the host cell, but only when these two response regulators are activated by phosphorylation, upon stress and high cellular density of the entire population of Bacilli. When phages are abundant, the lysogeny is triggered by the arbitrium system across the population of phages. Afterwards, phi3T is internalized under the form of the prophage. The Rapφ protein of the prophage might then confer an immediate advantage upon the lysogenized bacterium, enhancing the fitness of both the host and the prophage. Indeed, the inhibition of ComA-P and Spo0F-P as well as their target pathways linked to the production of antimicrobials and biofilm molecules might turn the lysogenized bacteria into cheaters. At high density of the lysogenized subpopulation, $Phr\varphi_{mature}$ is predicted to inhibit Rapφ, which, in turn, might alleviate ComA-P and Spo0F-P inhibition and repress the cheating phenotype.

MATERIALS AND METHODS

Detection of the Rapφ-Phrφ QS system

The QS receptor, Rapφ, was detected with Blastp (version 2.2.3+) (3) using, as a query, the protein sequences of reference RRNPP QS receptors and, as a target, all the protein sequences from the complete genomes of viruses that were available on the NCBI as of 18/07/19. The query was constructed based on the entries corresponding to RRNPP QS systems on the Quorumpeps database of QS signaling peptides (4) (NCBI accessions ANC26365.1 (ComR), AVD29695.1 (NprR), AAD41788.1 (PlcR), AAA65845.1 (PrgX), BAA11197.1 (TraA), ABQ95981.1 (Rgg), ABJ66531.1 (Rgg1358), NP_38125.1, NP_391550.1, NP_388259.1, NP_391519.1, NP_390460.2, NP_391626.1, NP_391910.1, NP_388565.2, NP_388382.1, NP_388164.1 (RapA to RapJ)). Rapφ was also independently detected with hmmsearch from the HMMER suite (version 3.2.1) (5), using, as query, a concatenation of the canonical HMM profiles of tetratricopeptide repeats that match the members of the RRNPP family of QS receptors (PF13181, PF13424, PF18710, PF18768 (Pfam models), SM00028, SM00530 (Smart models), 1.25.40.10 and 1.25.40.400 (CATH models), SSF48452 (Supfam models)). The QS pre-pro-peptide, Phrφ, has been detected as a signal peptide containing protein by SignalP (version 5.0b Linux x86_64) (6) with the key option '-org gram+', using as input the protein sequences encoded by the upstream and downstream genes of *rapφ.* Phrφ was further characterized as a Phr protein on the basis of the quality of its alignment with canonical Phr proteins (see below).

Evaluation of the genomic context of the *rapφ-phrφ* tandem of genes

The map of the Phi3T genome was produced with DNAplotter (release 1.11) (7), using as input the Genbank annotation file of the phi3T genome retrieved from the NCBI. The genes of phi3T highlighted as 'similar in SPbeta' were annotated as 'similar to SPBeta' in the annotation file. The functional annotation of the surrounding genes of the QS systems in Figure 1 relied either on the annotations present in the annotation file whenever they were

available, or were deduced from their constitutive domains, as detected by interproscan (version 5.36-75.0). The positions of intrinsic (or rho-independent) terminators in the genome of *Bacillus phage phi3T* were predicted by RNIE (8), configured with infernal (version 1.02) and launched independently with the '-genome' specific and the '-gene' sensitive modes and with a bitscore threshold of 14.

Multiple sequence alignment (MSA) of Rap and Phr proteins

The protein sequence of Rapφ was aligned with RapBA3 as well as with the Rap proteins from *B. subtilis strain 168* for which the structure has been resolved in the study of Gallego del Sol and Marina (9). This MSA was done with Muscle (version 3.8.31) (10) and visualized with Jalview version (2.10.5) (11) using the « % identity» colorcode to color the residues. The top annotation of the residues strictly corresponds to the one displayed by Gallego del Sol and Marina in their structure-guided MSA of *B. subtilis* Rap proteins. The whole protein sequence of Phrφ as well as its duplicated C-terminus have been aligned with PhrBA3, the duplicated C-terminus of PhrBA3, and all the canonical Phr proteins considered in Pottathil et al (12). The MSA was performed with Muscle, further manually adjusted, and visualized with Jalview (11) under the «Clustal» colorcode for residues (See http://www.jalview.org/help/html/colourSchemes/clustal.html*)*. The mature sequences of Phr were inferred manually from this MSA.

Mapping of RNA-Seq reads to the phi3T genome

Three replicates of RNA-Seq reads sequenced by Erez et al. (1) after 20min of infection of Bacillus subtilis strain BEST7003 by phi3T were retrieved (Sample accessions SAMEA24223918, SAMEA24226918 and SAMEA24229918) from the European Nucleotide Database. For each replicate, the reads were trimmed using fastx_clipper with default parameters (13). Then, we classically aligned the reads to the phi3T genome (NCBI accession KY030782) with Bowtie (14), allowing only one nucleotide misalignment and considering only the best alignment of each mapped read. The Integrative Genomics Viewer

was used to visualize both the coverage profile and the alignment of the reads to the *rapφ-phrφ* locus (15).

Identification of Rap-Phr systems in Bacillus' prophages

First, homologs of Rapφ were searched with Blastp (version 2.2.3+) (3) against all the protein sequences encoded by the complete genomes of Firmicutes available on the NCBI. A homolog was defined as a protein with no less than 30% sequence identity with a query protein, over more than 80% mutual coverage. The Rap homologs found by Blastp at each iteration were allowed to serve as queries in a next iteration, as long as the remote homologs exhibit 80% mutual coverage with Rapφ. Then, very stringent thresholds were applied to retain the Rap homologs that (i) harbor TPR repeats (E-value < 10^{-5} for at least one of the TPR HMM models cited above), (ii) do not have a DNA binding domain since Rap is not a transcription factor (E-value < 10^{-5} for any of the CATH 1.10.260.40, PFAM PF011381, SupFam SSF47413 and SMART SM00530 HMM models), (iii) are followed, on the same DNA strand and with an intergenic distance < 150bp, by a gene encoding a Phr protein, as defined by a protein of length between 15aa and 65aa and yielding a SEC-secretion likelihood score > 0.25 according to SignalP-5.0 (6). Next, for all the genomes identified as encoding at least one Rap-Phr system, the Phaster database (16) was queried to identify whether these genomes are composed of known prophage regions. At the time of the analysis, it was impossible to submit new queries to Phaster due to maintenance of the server, but this information was available for the genomes that had previously been queries by Phaster's users. If the coordinates of a *rap-phr* system fell within the coordinates of a prophage predicted by Phaster, the QS system was assessed as belonging to a prophage. The detailed results (with scores and NCBI IDs) of this analysis are given in Table S2.

Sequence similarity network of the Rap proteins

The sequence similarity network was built based on the output of the Blastp "all versus all" of the 2361 Rap proteins identified as forming a Rap-Phr QS system. An edge between two

nodes was drawn if the sequence identity between two Rap proteins was > 30% over more than 80% mutual coverage. The network was visualized with the Cytoscape software (17).

REFERENCES

1. Erez Z, Steinberger-Levy I, Shamir M, Doron S, Stokar-Avihail A, Peleg Y, et al. Communication between viruses guides lysis-lysogeny decisions. Nature. 2017;541(7638):488–93.
2. Even-Tov E, Omer Bendori S, Pollak S, Eldar A. Transient Duplication-Dependent Divergence and Horizontal Transfer Underlie the Evolutionary Dynamics of Bacterial Cell–Cell Signaling. Gore J, editor. PLOS Biol. 2016 Dec 29;14(12):e2000330.
3. Altschul SF, Gish W, Miller W, Myers EW, Lipman DJ. Basic local alignment search tool. J Mol Biol. 1990 Oct 5;215(3):403–10.
4. Wynendaele E, Bronselaer A, Nielandt J, D'Hondt M, Stalmans S, Bracke N, et al. Quorumpeps database: chemical space, microbial origin and functionality of quorum sensing peptides. Nucleic Acids Res. 2013 Jan 1;41(D1):D655–9.
5. Eddy SR. Accelerated Profile HMM Searches. Pearson WR, editor. PLoS Comput Biol. 2011 Oct 20;7(10):e1002195.
6. Almagro Armenteros JJ, Tsirigos KD, Sønderby CK, Petersen TN, Winther O, Brunak S, et al. SignalP 5.0 improves signal peptide predictions using deep neural networks. Nat Biotechnol. 2019 Apr 18;37(4):420–3.
7. Carver T, Thomson N, Bleasby A, Berriman M, Parkhill J. DNAPlotter: circular and linear interactive genome visualization. Bioinformatics. 2009 Jan 1;25(1):119–20.
8. Gardner PP, Barquist L, Bateman A, Nawrocki EP, Weinberg Z. RNIE: genome-wide prediction of bacterial intrinsic terminators. Nucleic Acids Res. 2011 Aug 1;39(14):5845–52.
9. Gallego del Sol F, Marina A. Structural Basis of Rap Phosphatase Inhibition by Phr Peptides. Laub MT, editor. PLoS Biol. 2013 Mar 19;11(3):e1001511.
10. Edgar RC. MUSCLE: multiple sequence alignment with high accuracy and high throughput. Nucleic Acids Res. 2004;32(5):1792–7.
11. Waterhouse AM, Procter JB, Martin DMA, Clamp M, Barton GJ. Jalview Version 2--a multiple sequence alignment editor and analysis workbench. Bioinformatics. 2009 May 1;25(9):1189–91.
12. Pottathil M, Lazazzera BA. The extracellular PHR peptide-Rap phosphatase signaling circuit of bacillus subtilis. Front Biosci. 2003 Jan 1;8(4):913.
13. FASTX-Toolkit [Internet]. [cited 2020 Aug 14]. Available from: http://hannonlab.cshl.edu/fastx_toolkit/
14. Langmead B, Trapnell C, Pop M, Salzberg SL. Ultrafast and memory-efficient alignment of short DNA sequences to the human genome. Genome Biol. 2009 Mar 4;10(3):R25.
15. Robinson JT, Thorvaldsdóttir H, Winckler W, Guttman M, Lander ES, Getz G, et al. Integrative genomics viewer. Nat Biotechnol. 2011 Jan 1;29(1):24–6.
16. Arndt D, Grant JR, Marcu A, Sajed T, Pon A, Liang Y, et al. PHASTER: a better, faster version

of the PHAST phage search tool. Nucleic Acids Res. 2016;44(Web Server issue):W16.

17. Shannon P, Markiel A, Ozier O, Baliga NS, Wang JT, Ramage D, et al. Cytoscape: a software environment for integrated models of biomolecular interaction networks. Genome Res. 2003 Nov;13(11):2498–504.
